# Autophagy, Acute Pancreatitis and the Metamorphoses of a Trypsinogen-Activating Organelle

**DOI:** 10.3390/cells11162514

**Published:** 2022-08-12

**Authors:** Svetlana Voronina, Michael Chvanov, Francesca De Faveri, Ulrike Mayer, Tom Wileman, David Criddle, Alexei Tepikin

**Affiliations:** 1 Department of Molecular Physiology and Cell Signalling, University of Liverpool, Liverpool L69 3BX, UK; 2 Biomedical Research Centre, School of Biological Sciences, University of East Anglia, Norwich NR4 7TJ, UK; 3 Quadram Institute Bioscience and Norwich Medical School, University of East Anglia, Norwich NR4 7UQ, UK

**Keywords:** acute pancreatitis, ATG8, autophagy, CASM, cholecystokinin, endocytic vacuole, LAP, LAP-like non-canonical autophagy, LC3, LC3-associated phagocytosis, LNCA, non-canonical autophagy, pancreatic acinar cell, trypsin, trypsinogen, zymogen granule

## Abstract

Recent studies have highlighted the importance of autophagy and particularly non-canonical autophagy in the development and progression of acute pancreatitis (a frequent disease with considerable morbidity and significant mortality). An important early event in the development of acute pancreatitis is the intrapancreatic activation of trypsinogen, (i.e., formation of trypsin) leading to the autodigestion of the organ. Another prominent phenomenon associated with the initiation of this disease is vacuolisation and specifically the formation of giant endocytic vacuoles in pancreatic acinar cells. These organelles develop in acinar cells exposed to several inducers of acute pancreatitis (including taurolithocholic acid and high concentrations of secretagogues cholecystokinin and acetylcholine). Notably, early trypsinogen activation occurs in the endocytic vacuoles. These trypsinogen-activating organelles undergo activation, long-distance trafficking, and non-canonical autophagy. In this review, we will discuss the role of autophagy in acute pancreatitis and particularly focus on the recently discovered LAP-like non-canonical autophagy (LNCA) of endocytic vacuoles.

## 1. Introduction 

The exocrine pancreas secretes digestive enzymes, precursors of digestive enzymes (termed zymogens), and bicarbonate-rich fluid. Zymogens and digestive enzymes are packaged in the secretory granules of pancreatic acinar cells and secreted by exocytosis (reviewed in [1,2]). Pancreatic acinar cells are polarised with apical regions bordering the lumen, continuous with the interior of pancreatic ducts, and the basal region adjacent to the interstitium (interspersed with blood vessels and nerves) [3]. Under physiological conditions zymogen granules are concentrated near the apical region of the acinar cells and the exocytotic secretion involves the fusion of zymogen granules with the apical (also termed luminal) plasma membrane [3,4]. The surface area of the apical/luminal plasma membrane is small and restricted [5]. The cells are capable of compound exocytosis, which allows the effective transfer of secreted material from multiple large secretory granules via the small apical/luminal membrane. This form of exocytosis is mediated by initial granule-to-plasma membrane fusion followed by granule-to-granule fusion (reviewed in [4]). Efficient endocytic membrane retrieval is essential for maintaining the secretory activity of the exocrine pancreas; however, the knowledge about post-exocytic membrane retrieval is limited. O. Larina and colleagues reported that F-actin is important for the apical endocytosis [6]. Post-exocytic structures (formed as a result of exocytosis of zymogen granules) are coated with F-actin [7,8] and retain actin at the early stages of endocytosis [9] indicating similarity to other forms of endocytosis (reviewed in [10]); actin dynamics are likely to be required for this type of membrane retrieval. Both “piecemeal endocytic retrieval” (i.e., disassembly of the postendocytic membrane by small endosomes)[11] and sudden retrieval of large postendocytic structures [12,13] have been described for apical endocytosis. It was reported that apical endocytosis is associated with cleavage of GP2 (pancreatic secretory granule membrane major glycoprotein 2) [14]. However, a later study challenged this notion [15]. In physiological conditions fluid-phase markers (and, most likely, retrieved post-exocytic membranes) are recycled to condensing vacuoles and later to novel secretory granules [16]. In pathophysiological conditions, fluid-phase endocytosis is redirected to autophagosomes and lysosomes [16]. Substances retrieved by membrane-associated endocytosis are targeted to autophagosomes and lysosomes [16]. 

Important secretagogues cholecystokinin (CCK) and acetylcholine (Ach) utilise Ca^2+^-signalling cascades to trigger the exocytosis of secretory granules in the acinar cells, (e.g., [17], reviewed in [1,4]). Other signalling cascades (notably cAMP signalling) also contribute to the exocytotic secretion of zymogens (reviewed in [18]). Both pancreatic acinar cells and pancreatic ductal cells participate in the fluid secretion [19,20]. Secretory products are delivered to the duodenum via the system of pancreatic ducts. In physiological conditions activation of zymogens occurs in the intestine and involves the enterokinase-dependent activation of trypsinogen with the downstream activation of other zymogens (reviewed in [21]). Early events in the development of acute pancreatitis include aberrant intrapancreatic activation of trypsinogen, i.e., formation of trypsin [22,23,24,25,26,27,28]. Both intracellular [23,24,29,30,31] and extracellular (in pancreatic ducts [28]) mechanisms of pancreatic trypsinogen activation have been reported. 

For many years, trypsinogen activation has been considered an important initiating and contributing factor in the development of acute pancreatitis—starting from a clinical hypothesis, put forward at the end of the 19th century [32]. This notion is consistent with later characterised pancreatitis-inducing mutations, associated with increased trypsinogen activation, increased trypsin stability and reduced trypsin inhibition (reviewed in [33,34,35]). However, the significance of trypsin activation to acute pancreatitis was recently challenged in the study by R. Dawra and colleagues from A. Saluja’s laboratory that reported the development of acute pancreatitis in mice with knocked out main form of trypsinogen (trypsinogen isoform 7) [36]. This paper stipulated that there was no resolvable effect of the trypsinogen knockout on the inflammatory response [36]. Nevertheless, several studies suggested that trypsinogen activation in pancreatic acinar cells could be sufficient to initiate acute pancreatitis [37,38,39]. The apparent contradiction can probably be reconciled assuming that trypsinogen activation is not the only damaging cellular mechanism involved in initiating acute pancreatitis; the redundancy and plurality of activating mechanisms is a notion vigorously discussed in this research field. Other identified damaging mechanisms include activation of calcineurin [40,41], activation of calpains [42,43], NF-κB activation [44], mitochondrial damage [45,46,47,48], redirection of exocytosis (from apical to basolateral region) [49,50] and aberrant autophagy [51]. Notably, numerous interactions between these damaging mechanisms have been reported, (e.g., [52,53,54]). The putative role of aberrant/disrupted autophagy is the focus of this review. 

Autophagy is an important degradative pathway. There are several autophagy subtypes including microautophagy, chaperone-mediated autophagy, and macroautophagy (canonical and non-canonical) [55,56,57]. This review will primarily discuss the role of macroautophagy (this will be referred to simply as “autophagy” in the text below). Canonical autophagy involves the formation of a double membrane phagophore engulfing the target structure; this is followed by the fusion of phagophore ends producing an autophagosome. The outer membrane of the autophagosome later interacts with the lysosome forming an autolysosome. The lysosomal hydrolases digest the inner membrane and the engulfed cargo. The breakdown products, (e.g., amino acids and peptides) are moved from the lumen of the organelle into the cytosol by lysosomal transporters and reutilised by the cell (recently reviewed in [58]). Canonical macroautophagy utilises several defined autophagy-related proteins (ATG proteins) [57,59], including ATG5, ATG7, ATG16, and ATG8/LC3 which will be mentioned in this review. Starvation, damaged organelles, and accumulation of proteins resistant to proteasomal degradation are prominent inducers of the canonical autophagy [60,61,62]. The molecular mechanisms initiating canonical autophagy usually include Unc-51 like autophagy activating kinase 1 and 2 (ULK1/2) (reviewed in [63]). An important step in canonical autophagy is ATG8/LC3 conjugation to the autophagosome membrane. This process requires ATG5, ATG7, ATG12, and notably ATG16L. The complex of these ATG proteins facilitates ATG8/LC3 lipidation (covalent binding to phosphatidylethanolamine (reviewed in [64])). ATG8/LC3 is a frequently used marker for studies of autophagy [56,65,66]. 

There are several subtypes of non-canonical autophagy in which some of the components, processes, and structural elements pertinent to canonical autophagy are omitted. LC3-associated phagocytosis (LAP) ([67,68] reviewed in [69]) and Rab9-mediated autophagy [70] are prominent examples of such non-canonical autophagy. Rab9-mediated autophagy is perhaps the most “non-canonical” form of autophagy. It can operate in cells with knocked out notable ATG proteins like ATG5 and ATG7 [70]. This form of non-canonical autophagy does not require ATG8/LC3 but is regulated by ULK1 [70]. LAP is another prominent recently discovered form of non-canonical autophagy [67,68,71]. This process does not require ULK1/2 but requires ATG8/LC3 conjugation and involves ATG5 and ATG7 [67]. However, within the framework of LAP, ATG8/LC3 conjugates to the single membrane of the phagosome (reviewed in [72]). Notably, in this case, ATG8/LC3 can bind to both phosphatidylethanolamine and phosphatidylserine [73,74]. Different domains of ATG16L are involved in LAP and canonical autophagy; the WD repeat-containing C-terminal domain (WD CT) of ATG16L1 is essential for LAP but dispensable for canonical autophagy [75,76]. It was later found that LAP is just one representative of a significant class of cellular processes involving single membrane ATG8/LC3 conjugation including ATG8/LC3 conjugations to structures formed as a result of entosis [77], micropinocytosis [77] as well as to vacuoles induced by osmotic imbalance [78]. The term CASM (conjugation of ATG8 to single membranes) was introduced to describe these related phenomena [73]. LAP could be considered as a prominent and the first-identified subtype of CASM. Several studies indicated that inhibitors of the vacuolar H^+^ ATPase (V-ATPase) can effectively block single membrane ATG8/LC3 conjugation [9,78,79]. A recent investigation reaffirmed the important role played by V-ATPases in the LC3 conjugation to single membranes [80]; interaction of ATG8/LC3 conjugating machinery with V-ATPases represents the defining property of CASM [80]. 

The recent discoveries of fundamental molecular mechanisms shaping canonical and non-canonical autophagy have influenced our understanding of the role played by autophagy in the physiology and pathophysiology of the exocrine pancreas, and particularly its role in pancreatitis. 

## 2. Autophagy in Pancreatitis 

Autophagosomes, lysosomes, and autolysosomes are amongst the organelles suggested to mediate trypsinogen activation pertinent to acute pancreatitis (reviewed in [81]). Trypsinogen can undergo autoactivation, however, this process is unlikely to make a dominant contribution to the trypsin formation in experimental acute pancreatitis [25,26,82]. Furthermore, there is reasonably strong evidence indicating that trypsinogen is not activated in “normal” secretory granules, (e.g., [24]). In the second half of the 20th century, several studies suggested the importance of lysosomal hydrolases in the trypsinogen activation and initiation of acute pancreatitis [83,84,85,86]. The co-localisation theory of trypsinogen activation was introduced (reviewed in [87,88]). Within the framework of this theory “co-localisation of lysosomal hydrolases with digestive enzyme zymogens plays a critical role in permitting the intracellular activation of digestive enzymes that leads to acinar cell injury and pancreatitis” [88]. Particular attention was given to the activating role of cathepsin B, which is present in lysosomes and autolysosomes. A conceptually important study by W. Halangk and colleagues reported a significant reduction (but, notably, not complete elimination) of trypsinogen activation in pancreatic acinar cells from cathepsin B-deficient mice [25]. This was accompanied by a substantial reduction in the severity of experimental acute pancreatitis (particularly for the early time points of the disease development) in the cathepsin B knockout mice. These and other experiments, (e.g., [26]) provided further support for the co-localisation hypothesis. Several studies identified trypsinogen activation (presence of trypsin) in intracellular vacuoles, (e.g., [13,24,30,89]). 

The nature of the organelle serving as a cauldron for the mixing of lysosomal hydrolases and zymogens has been extensively debated. M. Steer and colleagues suggested that co-localisation occurs in “abnormal condensing vacuoles” [87]. Cathepsin B is a major lysosomal protease, also present in the secretory granules under physiological conditions [90,91]; therefore, changes in the environment in the secretory granule could potentially lead to trypsinogen activation. Endocytic vacuoles are formed following aberrant exocytosis/endocytosis of the secretory granules [13] and are likely to retain both cathepsin B and trypsinogen. This could be sufficient for the trypsinogen activation in this organelle (see next section for further discussion and illustration of this hypothetical mechanism). An elegant ultrastructural study by J. Resau and colleagues characterised the formation of crinophagic vacuoles by lysosomal engulfment of zymogen granules and by direct fusion of the granules with lysosomes [92]; the authors suggested crinophagy as a possible mechanism of the co-localisation and activation of zymogens [92]. Hashimoto and colleagues suggested a major role of autophagy and autolysosomes in the trypsinogen activation [93]. In this study, the authors generated mice with conditional knockout of ATG5 in pancreatic acinar cells [93] and reported reduced severity of experimental acute pancreatitis in these animals as well as reduced trypsinogen activation [93]. These results indicated an initiating role of autophagy in acute pancreatitis. On the other hand, a study by O. Mareninova and colleagues described impairment of autophagy in conditions of acute pancreatitis and suggested that both vacuolisation and trypsinogen activation are the consequences of impaired/retarded autophagy [51]. Notably, this study also indicated the role of ATG5 in the activation of trypsinogen, implying a complex and multifaceted role of autophagy in trypsin/trypsinogen processing and disease initiation [51] (reviewed and discussed in [81]). A later study from the same group suggested a mechanism for autophagic flux impairment in the acute pancreatitis [94]. The completion of autophagy and the degradation of the cargo requires interaction between autophagosomes and lysosomes. Lysosome-associated membrane proteins (LAMPs) are important regulators of lysosomal function and biogenesis (reviewed in [95,96]). Depletion of LAMP1 and LAMP2 during acute pancreatitis was reported and suggested as the mechanism responsible for the disruption of the autophagic flux, vacuolisation, and progression of acute pancreatitis [94]. Depletion of TFEB (transcription factor of EB) is another mechanism responsible for reduced autophagy in the acute pancreatitis [97]. TFEB is the master regulator of lysosomal biogenesis, and also induces transcription of several genes involved in autophagy (recently reviewed in [98,99]). TFEB is rapidly degraded in the caerulein model of acute pancreatitis resulting in an insufficient autophagy [97]. Genetic ablation of this transcription factor increased the severity of experimental acute pancreatitis [97,100], whilst TFEB overexpression was protective [100]. Furthermore, decreased nuclear TFEB was associated with the human pancreatitis [97].

GFP-LC3 is an extensively used marker of autophagy. GFP-LC3 mice provide a valuable research model for studies of autophagy in primary cells and tissues (including pancreas) [65]. It was however noted that pancreatic autophagic flux in GFP-LC3 mice is altered (with increased levels of lipidated LC3 in the pancreas). Furthermore, experimental acute pancreatitis in GFP-LC3 mice was more severe than in wild-type animals. These experiments confirmed the importance of autophagy for acute pancreatitis and suggested caution in interpreting the results of experimental pancreatitis models utilising GFP-LC3 mice (for further details see [101]). 

An interesting mechanism, reported recently by H. Gaisano’s laboratory, involves the depletion of Syntaxin 2 (STX-2) in the experimental acute pancreatitis [49]. The depletion of STX-2 (in STX-2 KO mice) increased the binding of ATG16L1 to clathrin with the consequent impairment of autophagic flux and increased severity of experimental acute pancreatitis [49]. Notably, depletion of another SNARE protein, SNAP23, decreased the severity of acute pancreatitis by reducing the number of trypsinogen-activating autolysosomes [50]. Cytoprotective and homeostatic roles of autophagy in the exocrine pancreas have received considerable support in several recent studies. In particular, K. Diakopoulos and colleagues discovered that pancreas-specific depletion of ATG5 results in the development of pancreatitis in transgenic animals [102]. In the same vein, pancreas-specific knockout of ATG7 resulted in the development of spontaneous pancreatitis [103,104], suggesting a cytoprotective role of autophagy in the exocrine pancreas [103,104]. The study by Xia and colleagues confirmed that LAMP2 depletion in the pancreas also resulted in the increased severity of LPS-induced pancreatic damage [104]. Models of pancreatitis relying on the genetic ablation of autophagy/lysosomal pathways are discussed in a recent review [105].

The presence of zymogen granules in autophagosomes has been reported in several studies, (e.g., [51,65,93,106]). Potentiation of autophagy provided an alternative approach for revealing its role in acute pancreatitis. Constitutive expression of VMP1-GFP in pancreatic acinar cells of the Ela1-VMP1 mouse potentiated autophagy and engulfment of zymogen granules with double-membraned autophagosomes; this process was termed zymophagy [107]. The Ela1-VMP1 mice with enhanced autophagy demonstrated protection against caerulein—induced acute pancreatitis (including reduced trypsinogen activation) [107]. Finally, the protective effect of canonical autophagy was stipulated in a recent study examining the relationship between canonical autophagy and the Rab9-mediated non-canonical autophagy [108]. This study reported antagonistic relationships between the two forms of autophagy; it described Rab-9 depletion in conditions of experimental acute pancreatitis and suggested that this depletion potentiates canonical autophagy and its protective effects [108].

## 3. Endocytic Vacuoles: Actination, Rupture and LAP-like Non-Canonical Autophagy

**Figure 1:**
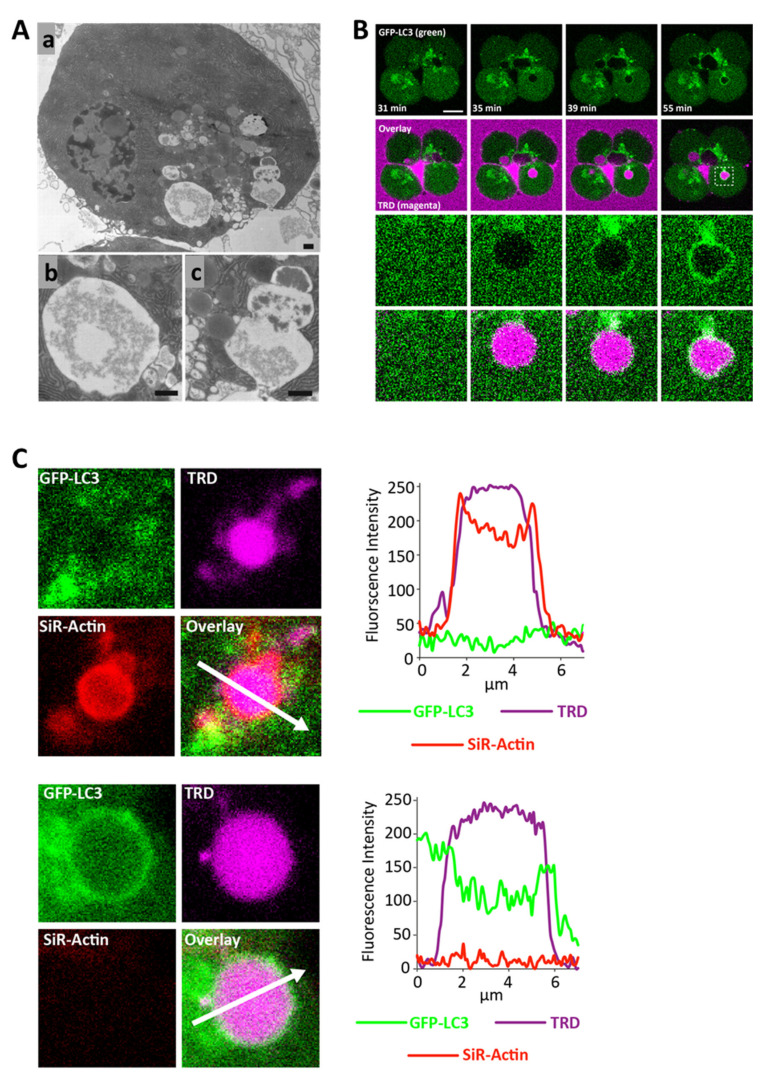
Formation, actination and LC3 conjugation of endocytic vacuoles. (**A**) Electron microscopy of endocytic vacuole and postendocytic structure (adapted with modifications from [13], copyright 2007 by The National Academy of Sciences of the USA). All scale bars correspond to 0.5 µm. (**Aa**) Pancreatic acinar cell stimulated with a high concentration of CCK (10 nM). (**Ab**) Enlarged fragment containing endocytic vacuole. (**Ac**) Post-endocytic structure, (i.e., precursor of an endocytic vacuole) formed as a result of compound exocytosis. Note the gradient of secretory material in the post-endocytic structure and the adjacent zymogen granules. (**B**) Formation and LAP-like non-canonical autophagy of an endocytic vacuole (adapted with modifications from [9]. Endocytic vacuoles, formed in pancreatic acinar cells as a result of CCK stimulation, are revealed by endocytosed Texas Red dextran (shown by the magenta colour in the figure). The cells were isolated from GFP-LC3 transgenic mice (GFP-LC3 is represented by the green colour in the figure). The upper two rows show the events in a cluster composed of four cells. Scale bar corresponds to 10 µm. The fragment (outlined by dash lines on the right overlay image) is shown in the two lower rows. Note the rapid LC3 conjugation to the endocytic vacuole. For further details see [9]. (**C**) Actination and LC3 conjugation of endocytic vacuoles formed in a CCK-stimulated pancreatic acinar cell (adapted with modifications from [9]). The cells were isolated from GFP-LC3 transgenic mice (GFP-LC3 is represented by the green colour in the figure). The endocytic vacuoles are revealed by endocytosed Texas Red dextran (shown by magenta colour in the figure). The F-actin is identified by SiR-actin staining (shown in red colour in the figure). The upper and lower panels represent fragments of confocal images shown in [9]. The upper panel depicts an actinated endocytic vacuole, which is coated with LC3. The lower panels show an LC3-conjugated endocytic vacuole, which is not actinated. The right parts of the panels show profiles of fluorescence intensities recorded along the corresponding white arrows. For further details see [9].

Studies from our laboratory characterised endocytic vacuoles in pancreatic acinar cells (Figure 1) and identified these organelles as the site of trypsinogen activation in the cells stimulated with the inducers of acute pancreatitis [9,13,109]. In experimental conditions the endocytic vacuoles can be identified by the presence of endocytosed membrane-impermeant fluorescent markers ([9,13,109], see Figure 1B,C). In this section we will discuss metamorphoses of endocytic vacuoles including recently discovered non-canonical autophagy of these organelles (revealed by LC3 conjugation [9], see Figure 1B,C). The formation of endocytic vacuoles is the earliest form of vacuolisation in the acinar cells subjected to inducers of acute pancreatitis taurolithocholic acid 3-sulfate (TLC-S) or a high concentration of CCK [9,109]; these vacuoles are clearly resolvable within 3 min from the beginning of the stimulation and remain the dominant form of cytosolic vacuoles for at least 1 h [9,109]. 

**Figure 2:**
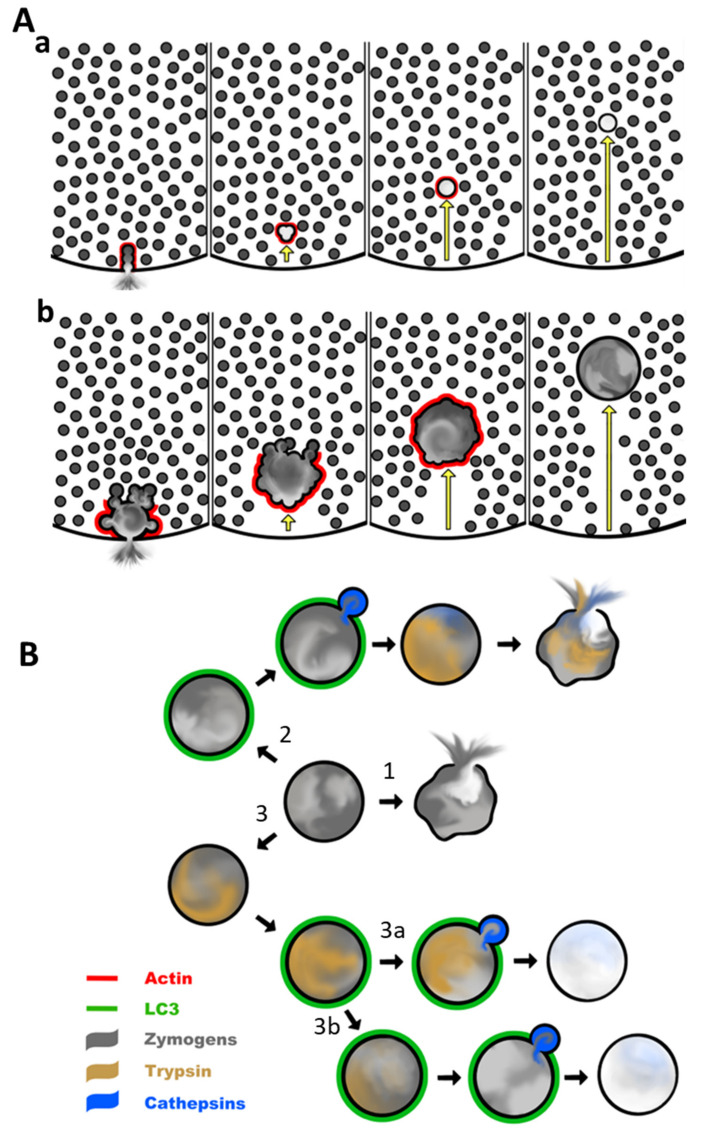
Simplified diagram illustrating hypothetical processes involving physiological compound exocytosis and excessive compound exocytosis leading to the formation of a large endocytic vacuole followed by its actination, LC3 conjugation, and trypsinogen activation. The opacity of the grey colour reflects the concentration of zymogens. The red colour represents polymerised actin, green colour indicates LC3 conjugation, blue colour indicates lysosomal cathepsins and dark yellow colour reflects intraorganellar activation of trypsinogen (formation of trypsin). (**A**) Early events involving formation, actination, and trafficking of post-exocytic structures and endocytic vacuoles. (**Aa**) Illustrates exocytosis and post-exocytic events in physiological conditions (mediated by moderate stimulation with physiological secretagogues). During a physiological response, only a few secretory granules participate in the compound exocytosis. The small volume of the post-exocytic structure allows nearly complete release of the zymogens into the lumen. Signals driving exocytosis are limited in amplitude and duration; actination of the post-exocytic structures is sufficiently fast to prevent excessive fusion. The small vacuole is trafficked away from the secretory region and disassembled. (**Ab**) Represents excessive compound exocytosis and formation of a large endocytic vacuole. These events are initiated by large concentrations of secretagogues or bile acids. Signals driving exocytosis (particularly Ca^2+^ signals) are strong and persistent. Actination of the post-exocytic organelle is not sufficiently fast and consequently a large number of secretory granules (up to 200) continue to fuse with the post-exocytic structure. The fusion pore closes trapping zymogens inside a large endocytic vacuole diluted by luminal fluid. Fusion of zymogen granules with the endocytic vacuole continues even after the closure of the fusion pore. The vacuole disconnects from the apical plasma membrane. The endocytic vacuole is finally completely actinated and fusion of secretory granules with the vacuole stops. The endocytic vacuole (containing a significant concentration of zymogens) is transported from the secretory region into the basolateral part of the cell and sheds its actin coat. (**B**) Illustration of the hypothetical evolution of the endocytic vacuole after it sheds actin (i.e., after the events shown in part Aa). Pathway **1** illustrates the putative process involving the rupture of an endocytic vacuole (before trypsinogen activation). Zymogens in this case are released into the cytosol and could potentially be activated in the cytosol. Pathway **2** demonstrates a putative role of LC3 conjugation, CASM/LNCA, and lysosomal hydrolases in activating trypsinogen with subsequent vacuolar rupture and the release of trypsin into the cytosol. Such a pathway is likely to cause cell damage. Pathway **3a** illustrates a putative protective role of CASM/LNCA against trypsin-induced damage. In this case, trypsinogen is activated (trypsin is formed) in the endocytic vacuole independently from LC3 conjugation; LC3 conjugation with the subsequent lysosomal fusion results in the destruction of the trypsin by lysosomal hydrolases. Pathway **3b.** Illustrates another putative protective mechanism involving lysosomal fusion. In this case, lysosomes repair the vacuolar membrane preventing the rupture of the vacuole and the release of trypsin (or trypsinogen) into the cytosol. It is conceivable that both protective mechanisms (shown on **3a** and **3b**) can operate simultaneously.

Endocytic vacuoles usually form in the apical region of the cell as the result of an aberrant compound exocytosis [13]. This involves excessive fusion of multiple zymogen granules during exocytosis ([13], see Figure 1A and Figure 2(Ab)); during this process the first granule fuses with the plasma membrane, and other granules undergo granule-to-granule fusion forming giant post-exocytic structures [8,13]. The next stages of the process include the closure of the fusion pore [11] and disconnection of the post-exocytic structure from the plasma membrane ([13], see Figure 1A and Figure 2(Ab)). The endocytic vacuole then migrates from the apical region (dominated by zymogen granules) to the basolateral part of the cell [13,109]. Trafficking of LC3-conjugated endocytic vacuoles from the secretory granule/apical region in the basolateral direction could be required for the processing of these organelles. It is conceivable that interaction with lysosomes and the formation of autolysosomes (see below) requires a specific topographical location of LC3-conjugated endocytic vacuoles outside of the granular region, (e.g., in proximity to the microtubule organising centre or simply in a region with sufficient density of lysosomes). The vacuoles can reach a diameter of 10 µm [9,13,109]. At the early stage of their formation and evolution, endocytic vacuoles are coated by polymerised actin, (i.e., actinated [9,109], see Figure 1C upper panel and Figure 2A). Actination of post-exocytic structures (also termed Ω-shapes [8]), starts very soon after the beginning of exocytosis and continues simultaneously with exocytosis [7,8]. It is possible that actination continues even after the disconnection of the vacuole from the plasma membrane. It is likely that polymerised actin plays an important “barrier” role [110] regulating secretion and preventing excessive fusion of zymogen granules. 

Figure 2A illustrates a hypothetical model in which there is competition between actination and fusion of zymogen granules, and its consequence for the retention of zymogens. Under physiological conditions Figure 2(Aa) short Ca^2+^ transients trigger exocytosis and fusion of only few secretory granules; actination prevents further fusion. Most of the zymogens, initially contained in the post-exocytic structure, are released via the fusion pore. The pore closes after a few minutes [11] and the small post-exocytic structure is disconnected from the plasma membrane [109]. This is trafficked away from the secretory granule region (further decreasing the probability of unwanted fusion with zymogen granules), loses its actin coat, and is eventually disassembled. Since most of the trypsinogen (and other zymogens) is released from the post-exocytic structure during exocytosis there is no possibility of trypsinogen activation inside the cell and the organelle. Notably P. Thorn and colleagues also described “piecemeal endocytic retrieval” of small membrane patches from the post-exocytic structures [11]; this process is also expected to prevent the retention of zymogens. 

The Figure 2(Ab) illustrates hypothetical pathophysiological scenarios. Strong and protracted Ca^2+^ signals generated in the acinar cells by the inducers of pancreatitis (reviewed in [111,112,113]) trigger exocytosis, characterised by excessive intergranular fusion. The rate of fusion exceeds the rate of actination so that polymerised actin only partially coats the post-exocytic structure and does not completely prevent aberrant fusion of zymogen granules. The fusion pore closes after a few minutes, trapping some of the zymogens in the endocytic vacuole, and the large endocytic vacuole disconnects from the plasma membrane [9,13,109]. Several zymogen granules fuse with the endocytic vacuole after the closure of the fusion pore (this fusion is a hypothetical phenomenon), the vacuole finally becomes completely actinated (preventing further fusions) and is trafficked vectorially from the secretory granule region in the basolateral direction [13,109]; trypsinogen (and presumably other zymogens trapped inside endocytic vacuole) are activated [9,13,109]. Actination and trafficking from the region populated with zymogen granules are likely to serve as protective mechanisms preventing excessive fusion of the endocytic vacuole with the zymogen granules and delivery of zymogens into the protease-activating endocytic vacuoles; these mechanisms are overwhelmed by the inducers of acute pancreatitis. Actination probably also plays a structural protective role; this possibility was suggested following experiments by M. Chvanov and colleagues that resolved rupture of vacuoles occurring via the fenestration in the actin coat [109]. Actination is a transient process; most of the endocytic vacuoles lose actin after only 20 min [9]. Importantly, the loss of actin is accompanied by LC3 conjugation to the surface of the endocytic vacuole ([9], see Figure 1C and Figure 2(Ab)), highlighting autophagy as the next step in the evolution of these organelles [9]. 

Following the initial observation of LC3 conjugation to the endocytic vacuoles we attempted to use a common experimental manoeuvre—apply the inhibitor of V-ATPase bafilomycin A1 to prevent the progression of autophagic flux and accumulate autophagosomes containing endocytic vacuoles for further studies. To our considerable surprise the LC3 conjugation to the endocytic vacuoles was completely inhibited by bafilomycin A1 suggesting that we were dealing with some form of non-canonical autophagy [9]. Our further experiments confirm this conclusion; LC3 conjugation to the endocytic vacuoles was strongly inhibited by another inhibitor of V-ATPase concanamycin A; the conjugation was insensitive to ULK1 inhibitors and required the WD40 domain of ATG16L1 [9]. Notably, correlative light (confocal fluorescence) and electron microscopy (CLEM) identified single-membrane conjugation of LC3 to the endocytic vacuoles [9]. The properties of this LC3 conjugation are similar to that recorded during LAP [67,68]. In particular, the conjugation also involved a single-membraned organelle (i.e., did not require phagophore formation). We termed the process involving LC3 conjugation to endocytic vacuoles LAP-like non-canonical autophagy (LNCA) [9,56]. Notably, unlike LAP, the observed LC3 conjugation to endocytic vacuoles is insensitive to antioxidants and is therefore unlikely to be induced by locally generated reactive oxygen species (ROS) (compare [71] and [9]). The LNCA in the acinar cells is probably a subtype of CASM; this conclusion is based on the common single-membrane LC3 conjugation for both processes and on the striking effect of V-ATPase inhibitors on LNCA [9] (the interaction of LC3-conjugating machinery with V-ATPases is a defining property of CASM [73,78,80]). Furthermore, both LNCA in the acinar cells and CASM require ATG16L1 with an intact C-terminal WD domain [9,75,76]. There is, however, an interesting peculiarity of LNCA in the acinar cell; it is inhibited by protonophores, (e.g., by Monensin) [9], whilst other forms of CASM can be induced by protonophores [78]. 

A remarkable property of LNCA is its speed; the earliest LC3 conjugated endocytic vacuoles have been observed within 10–20 min from the beginning of stimulation with supramaximal doses of CCK [9]. This contrasts with the recent observation that autophagy in acinar cells requires a few hours to develop [114]. The remarkable speed of LC3 conjugation to the endocytic vacuoles makes LNCA potentially relevant to early trypsinogen activation, resolvable within 10–20 min after the beginning of stimulation (which is too fast for canonical autophagy). Notably, the presence of trypsinogen-activating peptide (TAP) has been identified in the LC3-conjugated endocytic vacuoles [9]. It is conceivable that slower phagophore-mediated autophagy contributes to trypsinogen activation at later time points; in particular, LNCA in acinar cells should not have a kinetic advantage over canonical autophagy during the second peak of trypsinogen activation, observed in the caerulein model of acute pancreatitis[114]. The LNCA could be also relevant to early trypsin degradation (the early decrease in trypsin activity which is resolvable in pancreata after approximately 120 min from the beginning of stimulation) [25,114]. These putative contrasting effects of LNCA are illustrated in Figure 2B. This figure highlights the hypothetical fate of an endocytic vacuole starting from the time at which the vacuole loses its actin, (i.e., timewise this is a continuity of events shown in Figure 2A). Notably, Figure 2B includes a prominent recently observed phenomenon, namely rupture of an endocyclic vacuole [109] (the endpoints of pathways 1 and 2 are indicated in the figure). The rupture could be independent of the LC3 conjugation and trypsinogen activation. In this case, trypsinogen is released into the cytosol before LC3 conjugation and before trypsin is formed in the vacuole (see pathway 1 in Figure 2B; this could be followed by the cytosolic activation of zymogens). 

Another scenario is illustrated by pathway 2 in Figure 2B; LNCA could precipitate fusion with a lysosome, (i.e., formation of autolysosome), activation of trypsinogen by lysosomal hydrolases, (e.g., cathepsin B [25]) and finally rupture of the endocytic vacuole, releasing trypsin and cathepsins into the cytosol. This pathway illustrates a putative damage-inducing role of LNCA. The presence of TAP in LC3-conjugated endocytic vacuoles has been observed experimentally [9]; this strongly suggests the presence of trypsin in these organelles and is consistent with pathway 2. It is, however, also conceivable that LC3 conjugation plays a protective role. Such a hypothetical protective role is illustrated by pathways 3a and 3b in Figure 2B. In pathway 3a, activation of trypsinogen occurs independently of the LC3 conjugation. This could occur as a result of trypsinogen autoactivation (likely modulated by chymotrypsin C) [115] in the environment of the endocytic vacuole. An alternative mechanism is trypsinogen activation by cathepsin B, reported to co-localise with trypsinogen in secretory organelles of the intact acinar cells [90,91]. Following the aberrant endocytosis triggered by inducers of pancreatitis, cathepsin B could be retained in the endocytic vacuoles together with trypsinogen and contribute to the trypsin formation [13]. The role of the LC3 conjugation at this juncture is in initiating lysosomal fusion and the consequent destruction of trypsin and other digestive enzymes by the lysosomal hydrolases “sanitising” the content of the endocytic vacuole. Cathepsin L is a putative candidate for trypsin and trypsinogen cleavage [116] in this organelle and could potentially fulfil the protective role associated with pathway 3a. The balance between cathepsin B (trypsinogen activating protease) [25] and cathepsin L (protease degrading trypsinogen and trypsin) [116] could determine the dynamics of the trypsin levels in endocytic vacuoles. Notably, the presence of membrane-bound lysosomal proteins on endocytic vacuoles has been reported [13], suggesting the progression of endocytic vacuoles to autolysosomes. The sanitised content of the autolysosome, (i.e., the products of hydrolysis by lysosomal enzymes) could be exported into the cytosol by autolysosomal transporters and reutilised by the cell. Alternatively, the content of these organelles could be released from the cell via exocytosis. Indeed, the fusion of endocytic vacuoles with the plasma membrane and exocytosis has been observed [109]; however, we do not know the evolutionary stage of the fusing vacuoles or their content, (e.g., it could be zymogens, active proteases or the products of digestion). 

A further protective scenario is illustrated by pathway 3b in Figure 2B; it is similar to that shown in the 3a, except in this case both trypsinogen activation and trypsin degradation are assumed to be independent of LC3 conjugation and delivery of lysosomal hydrolases. The degradation of the trypsin in this pathway could occur as a result of autolysis modulated by chymotrypsin C [117] (the roles of chymotrypsin C in the activation and degradation are reviewed in [33]). Notably, after disconnection from the plasma membrane endocytic vacuoles undergo Ca^2+^ release associated with a substantial reduction in intraorganellar Ca^2+^ [13]; changes in Ca^2+^ concentration have been shown to modulate both trypsinogen autoactivation and the ability of chymotrypsin C to assist activation or promote degradation [115,117]. The dynamics of both processes could be guided by changes in the vacuolar environment (including Ca^2+^ changes). The putative protective role of LNCA and lysosomal fusion in this pathway (3b) is based on the known ability of lysosomes to “patch up”, (i.e., prevent rupture of) cellular membranes, (e.g., [118,119]). Specifically, this hypothetical mechanism was suggested by analogy with the role of lysosomes in preventing/repairing rupture of phagosomes (see [119] and associated commentary [120]). Such a stabilising action of lysosomes could allow an endocytic vacuole to retain its content until autolysis of trypsin and destruction of other digestive enzymes is completed. The specific role of LNCA in endocytic vacuoles is currently being investigated in our laboratory and the hypothetical diagram in Figure 2 at the moment contains more questions than answers. We hope that this research will expand our knowledge of the role of LNCA/CASM in acute pancreatitis and provide further information on the fundamental properties of this phenomenon and its initiating signals. 

The rich tapestry of canonical and non-canonical autophagy pathways has been recently characterised in the exocrine pancreas. These pathways activate with distinct kinetics and are likely to contribute to different stages of the initiation and progression of pancreatitis. The accumulated knowledge will provide a solid foundation to understand the role of autophagy in pancreatitis and hopefully facilitate the development of specific therapy. 

## Data Availability

Not applicable.

## References

[1] Cooley M.M., Jones E.K., Gorelick F.S., Groblewski G.E., Gorelick F.S., Wil-liams J.A. (2021). Pancreatic acinar cell protein synthesis, intracellular transport, and export. The Pancreas: Biology and Physiology.

[2] Chandra R., Liddle R.A., Gorelick F.S., Williams J.A. (2021). Regulation of pancreatic secretion. The Pancreas: Biology and Physiology.

[3] Longnecker D.S., Gorelick F.S., Williams J.A. (2021). Anatomy and histology of the pancreas. The Pancreas: Biology and Physiology.

[4] Gaisano H.Y., Dolai S., Takahashi T., Gorelick F.S., Williams J.A. (2021). Physiologic exocytosis in pancreatic acinar cells and pathologic fusion underlying pancreatitis. The Pancreas: Biology and Physiology.

[5] Bolender R.P. (1974). Stereological analysis of the guinea pig pancreas. I. Analytical model and quantitative description of nonstimulated pancreatic exocrine cells. J. Cell Biol..

[6] Larina O., Bhat P., Pickett J.A., Launikonis B.S., Shah A., Kruger W.A., Edwardson J.M., Thorn P. (2007). Dynamic regulation of the large exocytotic fusion pore in pancreatic acinar. Cells Mol. Biol. Cell.

[7] Jang Y., Soekmadji C., Mitchell J.M., Thomas W.G., Thorn P. (2012). Real-Time measurement of f-actin remodelling during exocytosis using lifeact-egfp transgenic animals. PLoS ONE.

[8] Nemoto T., Kojima T., Oshima A., Bito H., Kasai H. (2004). Stabilization of exocytosis by dynamic f-actin coating of zymogen granules in pancreatic acini. J. Biol. Chem..

[9] De Faveri F., Chvanov M., Voronina S., Moore D., Pollock L., Haynes L., Awais M., Beckett A.J., Mayer U., Sutton R. (2020). LAP-like non-canonical autophagy and evolution of endocytic vacuoles in pancreatic acinar cells. Autophagy.

[10] Chakrabarti R., Lee M., Higgs H.N. (2021). Multiple roles for actin in secretory and endocytic pathways. Curr. Biol..

[11] Thorn P., Fogarty K.E., Parker I. (2004). Zymogen granule exocytosis is characterized by long fusion pore openings and preservation of vesicle lipid identity. Proc. Natl. Acad. Sci. USA.

[12] Nemoto T., Kimura R., Ito K., Tachikawa A., Miyashita Y., Iino M., Kasai H. (2001). Sequential-replenishment mechanism of exocytosis in pancreatic acini. Nat. Cell Biol..

[13] Sherwood M.W., Prior I.A., Voronina S.G., Barrow S.L., Woodsmith J.D., Gerasimenko O.V., Petersen O.H., Tepikin A.V. (2007). Activation of trypsinogen in large endocytic vacuoles of pancreatic acinar cells. Proc. Natl. Acad. Sci. USA.

[14] Freedman S.D., Katz M.H., Parker E.M., Gelrud A. (1999). Endocytosis at the apical plasma membrane of pancreatic acinar cells is regulated by tyrosine kinases. Am. J. Physiol..

[15] Yu S., Hao Y., Lowe A.W. (2004). Effects of GP2 expression on secretion and endocytosis in pancreatic AR4-2J cells. Biochem. Biophys. Res. Commun..

[16] Lerch M.M., Saluja A.K., Runzi M., Dawra R., Steer M.L. (1995). Luminal endocytosis and intracellular targeting by acinar cells during early biliary pancreatitis in the opossum. J. Clin. Investig..

[17] Liang T., Dolai S., Xie L., Winter E., Orabi A.I., Karimian N., Cosen-Binker L.I., Huang Y.-C., Thorn P., Cattral M.S. (2017). Ex vivo human pancreatic slice preparations offer a valuable model for studying pancreatic exocrine biology. J. Biol. Chem..

[18] Albers T., Sabbatini M.E., Gorelick F.S., Williams J.A. (2021). Cyclic nucleotides as mediators of acinar and ductal function. The Pancreas: Biology and Physiology.

[19] Lee M.G., Kim Y., Jun I., Aoun J., Muallem S., Gorelick F.S., Williams J.A. (2021). Molecular mechanisms of pancreatic bicarbonate secretion. The Pancreas: Biology and Physiology.

[20] Ishiguro H., Yamaguchi M., Yamamoto A., Gorelick F.S., Williams J.A. (2021). Regulation of pancreatic fluid and electrolyte secretion. The Pancreas: Biology and Physiology.

[21] Mössner J. (2010). New Advances in cell physiology and pathophysiology of the exocrine pancreas. Dig. Dis..

[22] Gudgeon A.M., Heath D.I., Hurley P., Jehanli A., Patel G., Wilson C., Shenkin A., Austen B.M., Imrie C.W., Hermon-Taylor J. (1990). Trypsinogen activation peptides assay in the early predic-tion of severity of acute pancreatitis. Lancet.

[23] Hofbauer B., Saluja A.K., Lerch M.M., Bhagat L., Bhatia M., Lee H.S., Frossard J.L., Adler G., Steer M.L. (1998). Intraacinar cell activation of trypsinogen during caerulein-induced pancreatitis in rats. Am. J. Physiol. Content.

[24] Otani T., Chepilko S.M., Grendell J.H., Gorelick F.S. (1998). Codistribution of TAP and the granule membrane protein GRAMP-92 in rat caerulein-induced pancreatitis. Am. J. Physiol. Content.

[25] Halangk W., Lerch M.M., Brandt-Nedelev B., Roth W., Ruthenbuerger M., Reinheckel T., Domschke W., Lippert H., Peters C., Deussing J. (2000). Role of cathepsin B in intracellular trypsinogen activation and the onset of acute pancreatitis. J. Clin. Investig..

[26] van Acker G.J.D., Saluja A.K., Bhagat L., Singh V.P., Song A.M., Steer M.L. (2002). Cathepsin B inhibition prevents trypsinogen activation and reduces pancreatitis severity. Am. J. Physiol. Liver Physiol..

[27] Sendler M., Weiss F.U., Golchert J., Homuth G., van den Brandt C., Mahajan U.M., Par-tecke L.I., Doring P., Gukovsky I., Gukovskaya A.S. (2018). Ca-thepsin B-mediated Activation of Trypsinogen in Endocytosing Macrophages Increases Severity of Pancreatitis in Mice. Gastroenterology.

[28] Pallagi P., Venglovecz V., Rakonczay Z., Borka K., Korompay A., Ozsvari B., Judak L., Sahin–Toth M., Geisz A., Schnur A. (2011). Trypsin Reduces Pancreatic Ductal Bicarbonate Secretion by Inhibiting CFTR Cl− Channels and Luminal Anion Exchangers. Gastroenterology.

[29] Leach S.D., Modlin I.M., Scheele G.A., Gorelick F.S. (1991). Intracellular activation of digestive zymogens in rat pancreatic acini. Stimulation by high doses of cholecystokinin. J. Clin. Investig..

[30] Kruger B., Albrecht E., Lerch M.M. (2000). The role of intracellular calcium signaling in premature protease activation and the onset of pancreatitis. Am. J. Pathol..

[31] Raraty M., Ward J., Erdemli G., Vaillant C., Neoptolemos J.P., Sutton R., Petersen O.H. (2000). Calcium-dependent enzyme activation and vacuole formation in the apical granular region of pancreatic acinar cells. Proc. Natl. Acad. Sci. USA.

[32] Chiari H. (1896). Über die Selbstverdauung des menschlichen Pankreas. Z. Für Heilkunde..

[33] Hegyi E., Sahin-Toth M. (2017). Genetic Risk in Chronic Pancreatitis: The Trypsin-Dependent Path-way. Dig. Dis. Sci..

[34] Mayerle J., Sendler M., Hegyi E., Beyer G., Lerch M.M., Sahin-Toth M. (2019). Genetics, Cell Biology, and Pathophysiology of Pancreatitis. Gastroenterology.

[35] Whitcomb D.C. (2010). Genetic Aspects of Pancreatitis. Annu. Rev. Med..

[36] Dawra R., Sah R.P., Dudeja V., Rishi L., Talukdar R., Garg P., Saluja A.K. (2011). Intra-acinar trypsinogen activation mediates early stages of pancreatic injury but not inflammation in mice with acute pancreatitis. Gastroenterology.

[37] Geisz A., Sahin-Toth M. (2018). A preclinical model of chronic pancreatitis driven by trypsinogen autoactivation. Nat. Commun..

[38] Gaiser S., Daniluk J., Liu Y., Tsou L., Chu J., Lee W., Longnecker D.S., Logsdon C.D., Ji B. (2011). Intracellular activation of trypsinogen in transgenic mice induces acute but not chronic pancreatitis. Gut.

[39] Wang J., Wan J., Wang L., Pandol S.J., Bi Y., Ji B. (2022). Wild-Type Human PRSS2 and PRSS1R122H Cooperatively Initiate Spontaneous Hereditary Pancreatitis in Transgenic Mice. Gastroenterology.

[40] Muili K.A., Ahmad M., Orabi A.I., Mahmood S.M., Shah A.U., Molkentin J.D., Husain S.Z. (2012). Pharmacological and genetic inhibition of calcineurin protects against carbachol-induced pathological zymogen activation and acinar cell injury. Am. J. Physiol. Gastrointest. Liver Physiol..

[41] Muili K.A., Wang D., Orabi A.I., Sarwar S., Luo Y., Javed T.A., Eisses J.F., Mahmood S.M., Jin S., Singh V.P. (2013). Bile acids induce pancreatic acinar cell injury and pancreatitis by activating calcineurin. J. Biol. Chem..

[42] Weber H., Huhns S., Luthen F., Jonas L. (2009). Calpain-mediated breakdown of cytoskeletal proteins contributes to cholecystokinin-induced damage of rat pancreatic acini. Int. J. Exp. Pathol..

[43] Weber H., Jonas L., Huhns S., Schuff-Werner P. (2004). Dysregulation of the calpain-calpastatin system plays a role in the development of cerulein-induced acute pancreatitis in the rat. Am. J. Physiol. Gastrointest. Liver Physiol..

[44] Chen X., Ji B., Han B., Ernst S.A., Simeone D., Logsdon C.D. (2002). NF-kappaB activation in pancreas induces pancreatic and systemic inflammatory response. Gastroenterology.

[45] Baumgartner H.K., Gerasimenko J.V., Thorne C., Ferdek P., Pozzan T., Tepikin A.V., Petersen O.H., Sutton R., Watson A.J.M., Gerasimenko O.V. (2009). Calcium elevation in mitochondria is the main ca2+ requirement for mitochondrial permeability transition pore (mPTP) opening. J. Biol. Chem..

[46] Mukherjee R., Mareninova O.A., Odinokova I.V., Huang W., Murphy J., Chvanov M., Javed M.A., Wen L., Booth D.M., Cane M.C. (2016). Mechanism of mitochondrial permeability transition pore induction and damage in the pancreas: Inhibition prevents acute pancreatitis by protecting production of ATP. Gut.

[47] Criddle D.N., Murphy J., Fistetto G., Barrow S., Tepikin A.V., Neoptolemos J.P., Sutton R., Petersen O.H. (2006). Fatty acid ethyl esters cause pancreatic calcium toxicity via inositol trisphosphate receptors and loss of atp synthesis. Gastroenterology.

[48] Biczo G., Vegh E.T., Shalbueva N., Mareninova O.A., Elperin J., Lotshaw E., Gretler S., Lugea A., Malla S.R., Dawson D. (2018). Mitochondrial Dysfunction, Through Impaired Autophagy, Leads to Endoplasmic Reticulum Stress, Deregulated Lipid Metabolism, and Pancreatitis in Animal Models. Gastroenterology.

[49] Dolai S., Liang T., Orabi A.I., Holmyard D., Xie L., Greitzer-Antes D., Kang Y., Xie H., Javed T.A., Lam P.P. (2018). Pancreatitis-Induced Depletion of Syntaxin 2 Promotes Autophagy and Increases Basolateral Exocytosis. Gastroenterology.

[50] Dolai S., Takahashi T., Qin T., Liang T., Xie L., Kang F., Miao Y.-F., Xie H., Kang Y., Manuel J. (2021). Pancreas-specific SNAP23 depletion prevents pancreatitis by attenuating pathological basolateral exocytosis and formation of trypsin-activating autolysosomes. Autophagy.

[51] Mareninova O.A., Hermann K., French S.W., O’Konski M.S., Pandol S.J., Webster P., Erickson A.H., Katunuma N., Gorelick F.S., Gukovsky I. (2009). Impaired autophagic flux mediates acinar cell vacuole formation and trypsinogen activation in rodent models of acute pancreatitis. J. Clin. Investig..

[52] Gukovskaya A.S., Gorelick F.S., Groblewski G.E., Mareninova O.A., Lugea A., Anto-nucci L., Waldron R.T., Habtezion A., Karin M., Pandol S.J. (2019). Recent Insights into the Pathogenic Mechanism of Pancreatitis: Role of Acinar Cell Organelle Disorders. Pancreas.

[53] Petersen O.H., Gerasimenko J.V., Gerasimenko O.V., Gryshchenko O., Peng S. (2021). The roles of calcium and ATP in the physiology and pathology of the exocrine pancreas. Physiol. Rev..

[54] Habtezion A., Gukovskaya A.S., Pandol S.J. (2019). Acute Pancreatitis: A Multifaceted Set of Organelle and Cellular Interactions. Gastroenterology.

[55] Parzych K.R., Klionsky D.J. (2014). An Overview of Autophagy: Morphology, Mechanism, and Regulation. Antioxid. Redox Signal..

[56] Klionsky D.J., Abdel-Aziz A.K., Abdelfatah S., Abdellatif M., Abdoli A., Abel S., Abelio-vich H., Abildgaard M.H., Abudu Y.P., Acevedo-Arozena A. (2021). Guidelines for the use and inter-pretation of assays for monitoring autophagy (4th edition)(1). Autophagy.

[57] Mizushima N., Komatsu M. (2011). Autophagy: Renovation of cells and tissues. Cell.

[58] Rudnik S., Damme M. (2021). The lysosomal membrane—export of metabolites and beyond. FEBS J..

[59] Klionsky D.J., Cregg J.M., Dunn W.A., Emr S.D., Sakai Y., Sandoval I.V., Sibirny A., Subramani S., Thumm M., Veenhuis M. (2003). A unified nomenclature for yeast autophagy-related genes. Dev. Cell.

[60] Hurley J.H., Young L.N. (2017). Mechanisms of Autophagy Initiation. Annu. Rev. Biochem..

[61] Shaid S., Brandts C.H., Serve H., Dikic I. (2013). Ubiquitination and selective autophagy. Cell Death Differ..

[62] Sica V., Galluzzi L., Pedro J.M.B.-S., Izzo V., Maiuri M.C., Kroemer G. (2015). Organelle-Specific Initiation of Autophagy. Mol. Cell.

[63] Zachari M., Ganley I.G. (2017). The mammalian ULK1 complex and autophagy initiation. Essays Biochem..

[64] Martens S., Fracchiolla D. (2020). Activation and targeting of ATG8 protein lipidation. Cell Discov..

[65] Mizushima N., Yamamoto A., Matsui M., Yoshimori T., Ohsumi Y. (2004). In vivo analysis of autophagy in response to nutrient starvation using transgenic mice expressing a fluorescent autophagosome marker. Mol. Biol. Cell.

[66] Mizushima N., Yoshimori T., Levine B. (2010). Methods in Mammalian Autophagy Research. Cell.

[67] Martinez J., Almendinger J., Oberst A., Ness R., Dillon C.P., Fitzgerald P., Hengartner M.O., Green D.R. (2011). Microtubule-associated protein 1 light chain 3 alpha (LC3)-associated phagocytosis is required for the efficient clearance of dead cells. Proc. Natl. Acad. Sci. USA.

[68] Sanjuan M.A., Dillon C.P., Tait S.W., Moshiach S., Dorsey F., Connell S., Komatsu M., Tanaka K., Cleveland J.L., Withoff S. (2007). Toll-like receptor signalling in macrophages links the autophagy pathway to phagocytosis. Nature.

[69] Heckmann B.L., Boada-Romero E., Cunha L.D., Magne J., Green D.R. (2017). LC3-Associated Phagocytosis and Inflammation. J. Mol. Biol..

[70] Nishida Y., Arakawa S., Fujitani K., Yamaguchi H., Mizuta T., Kanaseki T., Komatsu M., Otsu K., Tsujimoto Y., Shimizu S. (2009). Discovery of Atg5/Atg7-independent alternative macroautophagy. Nature.

[71] Martinez J., Malireddi R.K., Lu Q., Cunha L.D., Pelletier S., Gingras S., Orchard R., Guan J.L., Tan H., Peng J. (2015). Molecular characterization of LC3-associated phagocytosis reveals distinct roles for Rubicon, NOX2 and autophagy proteins. Nat. Cell Biol..

[72] Heckmann B.L., Green D.R. (2019). LC3-associated phagocytosis at a glance. J. Cell Sci..

[73] Durgan J., Florey O. (2021). A new flavor of cellular Atg8-family protein lipidation-alternative conjugation to phosphatidylserine during CASM. Autophagy.

[74] Durgan J., Lystad A.H., Sloan K., Carlsson S.R., Wilson M.I., Marcassa E., Ulferts R., Webster J., Lopez-Clavijo A.F., Wakelam M.J. (2021). Non-canonical autophagy drives alternative ATG8 conjugation to phosphatidylserine. Mol. Cell.

[75] Rai S., Arasteh M., Jefferson M., Pearson T., Wang Y., Zhang W., Bicsak B., Divekar D., Powell P.P., Nauman R. (2018). The ATG5-binding and coiled coil domains of ATG16L1 maintain autophagy and tissue homeostasis in mice independently of the WD domain required for LC3-associated phagocytosis. Autophagy.

[76] Fletcher K., Ulferts R., Jacquin E., Veith T., Gammoh N., Arasteh J.M., Mayer U., Carding S.R., Wileman T., Beale R. (2018). The WD 40 domain of ATG 16L1 is required for its non-canonical role in lipidation of LC 3 at single membranes. EMBO J..

[77] Florey O., Kim S.E., Sandoval C.P., Haynes C.M., Overholtzer M. (2011). Autophagy machinery mediates macroendocytic processing and entotic cell death by targeting single membranes. Nat. Cell Biol..

[78] Florey O., Gammoh N., Kim S.E., Jiang X., Overholtzer M. (2015). V-ATPase and osmotic imbalances activate endolysosomal LC3 lipidation. Autophagy.

[79] Jacquin E., Leclerc-Mercier S., Judon C., Blanchard E., Fraitag S., Florey O. (2017). Pharmacological modulators of autophagy activate a parallel noncanonical pathway driving unconventional LC3 lipidation. Autophagy.

[80] Hooper K.M., Jacquin E., Li T., Goodwin J.M., Brumell J.H., Durgan J., Florey O. (2021). V-ATPase is a universal regulator of LC3-associated phagocytosis and non-canonical autophagy. J. Cell Biol..

[81] Gukovskaya A.S., Gukovsky I. (2012). Autophagy and pancreatitis. Am. J. Physiol. Gastrointest. Liver Physiol..

[82] Halangk W., Kruger B., Ruthenburger M., Sturzebecher J., Albrecht E., Lippert H., Lerch M.M. (2002). Trypsin activity is not involved in premature, intrapancreatic trypsinogen activation. Am. J. Physiol. Liver Physiol..

[83] Greenbaum L.M., Hirshkowitz A. (1961). Endogenous Cathepsin Activation of Trypsinogen in Extracts of Dog Pancreas. Exp. Biol. Med..

[84] Greenbaum L.M., Hirshkowitz A., Shoichet I. (1959). The activation of trypsinogen by cathepsin B. J. Biol. Chem..

[85] Saluja A.K., Donovan E.A., Yamanaka K., Yamaguchi Y., Hofbauer B., Steer M.L. (1997). Cerulein-induced in vitro activation of trypsinogen in rat pancreatic acini is mediated by cathepsin B. Gastroenterology.

[86] Figarella C., Miszczuk-Jamska B., Barrett A. (1988). Possible lysosomal activation of pancreatic zymogens. Activation of both human trypsinogens by cathepsin B and spontaneous acid. Activation of human trypsinogen 1. Biol. Chem. Hoppe-Seyler.

[87] Steer M.L., Meldolesi J., Figarella C. (1984). Pancreatitis. The role of lysosomes. Dig. Dis. Sci..

[88] van Acker G.J., Perides G., Steer M.L. (2006). Co-localization hypothesis: A mechanism for the intra-pancreatic activation of digestive enzymes during the early phases of acute pancreatitis. World J. Gastroenterol..

[89] Kruger B., Lerch M.M., Tessenow W. (1998). Direct detection of premature protease activation in living pancreatic acinar cells. Lab. Investig..

[90] Kukor Z., Mayerle J., Kruger B., Tóth M., Steed P.M., Halangk W., Lerch M.M., Sahin-Toth M. (2002). Presence of cathepsin b in the human pancreatic secretory pathway and its role in trypsinogen activation during hereditary pancreatitis. J. Biol. Chem..

[91] Tooze J., Hollinshead M., Hensel G., Kern H.F., Hoflack B. (1991). Regulated secretion of mature cathepsin B from rat exocrine pancreatic cells. Eur. J. Cell Biol..

[92] Resau J.H., Marzella L., Trump B.F., Jones R.T. (1984). Degradation of zymogen granules by lysosomes in cultured pancreatic explants. Am. J. Pathol..

[93] Hashimoto D., Ohmuraya M., Hirota M., Yamamoto A., Suyama K., Ida S., Okumura Y., Takahashi E., Kido H., Araki K. (2008). Involvement of autophagy in trypsinogen activation within the pancreatic acinar cells. J. Cell Biol..

[94] Mareninova O.A., Sendler M., Malla S.R., Yakubov I., French S.W., Tokhtaeva E., Vagin O., Oorschot V., Lullmann-Rauch R., Blanz J. (2015). Lysosome associated membrane proteins maintain pancre-atic acinar cell homeostasis: LAMP-2 deficient mice develop pancreatitis. Cell Mol. Gastroenterol. Hepatol..

[95] Schwake M., Schroder B., Saftig P. (2013). Lysosomal membrane proteins and their central role in physiology. Traffic.

[96] Eskelinen E.-L. (2006). Roles of LAMP-1 and LAMP-2 in lysosome biogenesis and autophagy. Mol. Asp. Med..

[97] Wang S., Ni H.M., Chao X., Wang H., Bridges B., Kumer S., Schmitt T., Mareninova O., Gukovskaya A., De Lisle R.C. (2019). Impaired TFEB-mediated lysosomal biogenesis promotes the development of pancreatitis in mice and is associated with human pancreatitis. Autophagy.

[98] Tan A., Prasad R., Lee C., Jho E.-H. (2022). Past, present, and future perspectives of transcription factor EB (TFEB): Mechanisms of regulation and association with disease. Cell Death Differ..

[99] Napolitano G., Ballabio A. (2016). TFEB at a glance. J. Cell Sci..

[100] Wang S., Ni H.-M., Chao X., Ma X., Kolodecik T., De Lisle R., Ballabio A., Pacher P., Ding W.-X. (2020). Critical Role of TFEB-Mediated Lysosomal Biogenesis in Alcohol-Induced Pancreatitis in Mice and Humans. Cell Mol. Gastroenterol. Hepatol..

[101] Mareninova O.A., Jia W., Gretler S.R., Holthaus C.L., Thomas D.D.H., Pimienta M., Dillon D.L., Gukovskaya A.S., Gukovsky I., Groblewski G.E. (2020). Transgenic expression of GFP-LC3 perturbs autophagy in exocrine pancreas and acute pancreatitis responses in mice. Autophagy.

[102] Diakopoulos K.N., Lesina M., Wörmann S., Song L., Aichler M., Schild L., Artati A., Romisch-Margl W., Wartmann T., Fischer R. (2015). Impaired autophagy induces chronic atrophic pancreatitis in mice via sex- and nutrition-dependent processes. Gastroenterology.

[103] Antonucci L., Fagman J.B., Kim J.Y., Todoric J., Gukovsky I., Mackey M., Ellisman M.H., Karin M. (2015). Basal autophagy maintains pancreatic acinar cell homeostasis and protein synthesis and prevents ER stress. Proc. Natl. Acad. Sci. USA.

[104] Xia L., Xu Z., Zhou X., Bergmann F., Grabe N., Büchler M.W., Neoptolemos J.P., Hackert T., Kroemer G., Fortunato F. (2020). Impaired autophagy increases susceptibility to endotoxin-induced chronic pancreatitis. Cell Death Dis..

[105] Gukovskaya A.S., Mareninova O.A., Ding W.-X., Habtezion A., Gukovsky I. (2021). Models of pancreatitis caused by genetic blockage of autophagy/lysosomal pathway. Pancreapedia: Exocrine Pancreas Knowledge Base.

[106] Willemer S., Kloppel G., Kern H.F., Adler G. (1989). Immunocytochemical and morphometric analysis of acinar zymogen granules in human acute pancreatitis. Virchows Arch. A Pathol. Anat. Histopathol..

[107] Grasso D., Ropolo A., Re A.L., Boggio V., Molejon M.I., Iovanna J.L., Gonzalez C.D., Urrutia R., Vaccaro M.I. (2011). Zymophagy, a novel selective autophagy pathway mediated by VMP1-USP9X-p62, prevents pancreatic cell death. J. Biol. Chem..

[108] Mareninova O.A., Dillon D.L., Wightman C.J.M., Yakubov I., Takahashi T., Gaisano H.Y., Munson K., Ohmuraya M., Dawson D., Gukovsky I. (2021). Rab9 Mediates Pancreatic Autophagy Switch from Canonical to Noncanonical, Aggravating Experimental Pancreatitis. Cell Mol. Gastroenterol. Hepatol..

[109] Chvanov M., De Faveri F., Moore D., Sherwood M.W., Awais M., Voronina S., Sutton R., Criddle D.N., Haynes L., Tepikin A.V. (2018). Intracellular rupture, exocytosis and actin interaction of endocytic vacuoles in pancreatic acinar cells: Initiating events in acute pancreatitis. J. Physiol..

[110] Muallem S., Kwiatkowska K., Xu X., Yin H.L. (1995). Actin filament disassembly is a sufficient final trigger for exocytosis in nonexcitable cells. J. Cell Biol..

[111] Petersen O.H., Tepikin A.V. (2008). Polarized calcium signaling in exocrine gland cells. Annu. Rev. Physiol..

[112] Chvanov M., Voronina S., Criddle D.N., Tepikin A.V. (2020). The role of Ca^2+^ signalling in the physiology and pathophysiology of exocrine pancreas. Curr. Opin. Physiol..

[113] Yule D.I., Gorelick F.S., Williams J.A. (2021). Calcium signaling in pancreatic acinar cells. The Pancreas: Biology and Physiology.

[114] Malla S.R., Krueger B., Wartmann T., Sendler M., Mahajan U.M., Weiss F.U., Thiel F.G., De Boni C., Gorelick F.S., Halangk W. (2019). Early trypsin activation develops independently of autophagy in caerulein-induced pancreatitis in mice. Experientia.

[115] Nemeth B.C., Wartmann T., Halangk W., Sahin-Toth M. (2013). Autoactivation of mouse trypsinogens is regulated by chymotrypsin C via cleavage of the autolysis loop. J. Biol. Chem..

[116] Wartmann T., Mayerle J., Kahne T., Sahin–Toth M., Ruthenbürger M., Matthias R., Kruse A., Reinheckel T., Peters C., Weiss F.U. (2010). cathepsin l inactivates human trypsinogen, whereas cathepsin l-deletion reduces the severity of pancreatitis in mice. Gastroenterology.

[117] Szmola R., Sahin-Toth M. (2007). Chymotrypsin C (caldecrin) promotes degradation of human cationic trypsin: Identity with Rinderknecht’s enzyme Y. Proc. Natl. Acad. Sci. USA.

[118] Reddy A., Caler E.V., Andrews N.W. (2001). Plasma membrane repair is mediated by ca^2+^-regulated exocytosis of lysosomes. Cell.

[119] Westman J., Walpole G.F.W., Kasper L., Xue B.Y., Elshafee O., Hube B., Grinstein S. (2020). Lysosome Fusion Maintains Phagosome Integrity during Fungal Infection. Cell Host Microbe.

[120] Onyishi C.U., Desanti G.E., May R.C. (2020). Plugging a Leak: How Phagosomes “Stretch” to Accommodate Pathogen Growth. Cell Host Microbe.

